# PM_2.5_ exposure as a risk factor for type 2 diabetes mellitus in the Mexico City metropolitan area

**DOI:** 10.1186/s12889-021-12112-w

**Published:** 2021-11-13

**Authors:** Olivia L. Chilian-Herrera, Marcela Tamayo-Ortiz, Jose L. Texcalac-Sangrador, Stephen J. Rothenberg, Ruy López-Ridaura, Martín Romero-Martínez, Robert O. Wright, Allan C. Just, Itai Kloog, Luis F. Bautista-Arredondo, Martha María Téllez-Rojo

**Affiliations:** 1 Homologous Normative Coordination, General Directorate, Mexican Social Security Institute, Mexico City, Mexico; 2 Occupational Health Research Unit, Mexican Social Security Institute, Av. Cuauhtémoc 330, Doctores, Cuauhtémoc, 06720 Mexico City, Mexico; 3 Department of Environmental Health, Center for Population Health Research, National Institute of Public Health, Cuernavaca, Morelos Mexico; 4 National Center for Disease Prevention and Control Programs, Mexico City, Mexico; 5 Center for Research in Surveys and Evaluation, National Institute of Public Health, Cuernavaca, Morelos Mexico; 6 Department of Environmental Medicine and Public Health, Icahn School of Medicine at Mount Sinai, New York, NY USA; 7 Department of Geography and Environmental Development, Ben-Gurion University of the Negev, Beer-Sheva, Israel; 8 Center for Nutrition and Health Research, National Institute of Public Health, Cuernavaca, Morelos Mexico

**Keywords:** PM_2.5_, Particulate matter, Air pollution, Diabetes mellitus, type 2, Mexico

## Abstract

**Background:**

Exposure to air pollution is the main risk factor for morbidity and mortality in the world. Exposure to particulate matter with aerodynamic diameter ≤ 2.5 μm (PM_2.5_) is associated with cardiovascular and respiratory conditions, as well as with lung cancer, and there is evidence to suggest that it is also associated with type II diabetes (DM). The Mexico City Metropolitan Area (MCMA) is home to more than 20 million people, where PM_2.5_ levels exceed national and international standards every day. Likewise, DM represents a growing public health problem with prevalence around 12%. In this study, the objective was to evaluate the association between exposure to PM_2.5_ and DM in adults living in the MCMA.

**Methods:**

Data from the 2006 or 2012 National Health and Nutrition Surveys (ENSANUT) were used to identify subjects with DM and year of diagnosis. We estimated PM_2.5_ exposure at a residence level, based on information from the air quality monitoring system (monitors), as well as satellite measurements (satellite). We analyzed the relationship through a cross-sectional approach and as a case - control study.

**Results:**

For every 10 μg/m^3^ increase of PM_2.5_ we found an OR = 3.09 (95% CI 1.17–8.15) in the 2012 sample. These results were not conclusive for the 2006 data or for the case - control approach.

**Conclusions:**

Our results add to the evidence linking PM_2.5_ exposure to DM in Mexican adults. Studies in low- and middle-income countries, where PM_2.5_ atmospheric concentrations exceed WHO standards, are required to strengthen the evidence.

## Introduction

Exposure to particulate matter with aerodynamic diameter ≤ 2.5 μm (PM_2.5_) is directly related to morbidity and mortality due to lung cancer, cardiovascular and respiratory conditions [1–4] however, recent evidence suggests that it is also associated with the incidence and prevalence of diabetes [5–9] but results remain inconclusive [10–17].

Experimental studies suggest that the biological mechanisms underlying the relation between PM_2.5_ and DM are related to: a) endothelial dysfunction [18] that precedes insulin resistance and the reduction of peripheral glucose uptake [19]; b) with stress in the endoplasmic reticulum, which results in insulin transductions [20], which in the long-term, impair insulin synthesis and cause apoptosis of pancreatic β cells [21] and c) a decrease of brown adipose tissue, and alterations in mitochondria [6, 19, 20, 22, 23].

Most of the epidemiological studies that found positive associations of DM with long-term PM_2.5_ exposures have been carried out in developed countries: Canada [24–26], United States [12, 27–29], Denmark [30], Saudi Arabia [31], and Italy [32], where average annual PM_2.5_ concentrations were lower than those commonly recorded in developing countries. Recently, four studies conducted in cities of China reported elevated exposure to PM_2.5_ concentrations. They found positive, statistically significant and consistent associations with DM, but not significantly greater than those observed in developed countries [33–36], suggesting more studies in other developing cities could contribute to clarify the relationship [9, 15, 37, 38]. Given a possible concentration-response relationship [5, 6, 39], it has also been suggested to evaluate prolonged periods of exposure (> 6 months) in countries where PM_2.5_ concentrations are usually higher than those already analyzed.

The heterogeneity of the results from epidemiological studies is also due to differences in exposure assessment methods and data sources, statistical methods [7, 36, 40], as well as the study design [38]. Data from government monitoring networks are the main source of information to assess exposure. However, in some cities the coverage by monitors is limited, resulting in inaccurate exposure estimates with increased measurement error [41, 42]. Therefore, some studies have used satellite data based on tele-detection of aerosol optical depth (AOD), to evaluate exposure at a higher resolution [43, 44]. Regarding study design, prevalence studies are more common in low-and-middle income countries [38], however the exposure estimation methods are usually based on exposure averages from previous years. In absence of cohort studies, nested case-control analyses considering diagnosis date are an alternative to indirectly analyze DM incidence.

In the Mexico City Metropolitan Area (MCMA), one of the five mega-cities in the world and home to more than twenty million people, public policies to improve air quality have had a positive impact on the reduction of air pollution. However, the levels of PM_2.5_ have slowed their decline to concentrations that still exceed the WHO recommendations (annual average < 10 μg/m^3^ and maximum 24 h < 25 μg/m^3^) [45]. Since the mid-2003 the MCMA air quality monitoring system measures atmospheric concentrations of PM_2.5_ hourly. Additionally, there is PM_2.5_ information determined by AOD measurements and calibrated with the monitoring system data since 2004. Considering undiagnosed DM, it is estimated that 11.5 million people in Mexico have the disease, and for 2040, this will increase to 20.6 million [46]. The prevalence of diagnosed DM in the country is 10.7% and about 13% in the MCMA [47]. Therefore, the objective of this study was to evaluate the association between PM_2.5_ exposure and DM prevalence in adult residents of the MCMA using two different PM_2.5_ assessment methods 1) monitoring system and 2) satellite measurements; and two epidemiological designs: 1) a cross-sectional analysis of the National Health and Nutrition Surveys (ENSANUT) years 2006 and 2012, and 2) a case-control analysis nested in the cross-sectional 2012 ENSANUT study.

## Methods

We used data from the ENSANUTs 2006 and 2012 that are independent cross-sectional surveys designed to estimate population parameters describing the health and nutrition conditions of people in each of the 32 federative entities of the Mexican Republic. The stratified, probabilistic and multi-stage cluster selection design (selection of basic geostatistical areas, homes and individuals) has been described in detail in previous studies [48, 49]. The samples of the ENSANUT 2006 and 2012 have national and state representativeness; our study population is a representative subsample from each of the surveys of the adult population (≥20 years old) residing in the municipalities of the State of Mexico and Mexico City that make up the MCMA. For the 2006 survey we included data for a total of *n* = 2275 persons (*n* = 193 with diabetes and *n* = 2082 without diabetes) representing *n* = 12,655,760 persons (*n* = 1,045,037 with diabetes and *n* = 11,610,723 without diabetes); for the 2012 survey we included data for a total of *n* = 2297 persons (*n* = 284 with diabetes and *n* = 2013 without diabetes) representing *n* = 13,731,902 persons (*n* = 1,547,262 with diabetes and *n* = 12,184,640 without diabetes). General health and demographic information for the ENSANUTs were collected through questionnaires applied by trained and standardized survey personnel.

### Analyses approach

We considered two analysis approaches based on the availability of exposure and outcome data, a cross-sectional and a case-control approach. The cross-sectional analyses approach considered the data for both the 2006 and 2012 ENSANUT as independent samples. We compared people with and without DM considering the average PM_2.5_ exposure of a year prior to the date of the respective survey. For the case-control approach we considered the average PM_2.5_ exposure for the two years prior to the date of DM diagnosis as a proxy for cumulative exposure. Our exposure data is from 2004 onward therefore, we used data only from the ENSANUT 2012 and included cases with a DM diagnosis from 2006 onward (using the 2006 survey data resulted in very small study population with few DM cases we could assign exposure to, since this meant we could only include cases that reported a diagnosis in 2006). The case-control study included *n* = 121 DM cases and *n* = 480 controls.

### Diabetes and other demographic information

We obtained self-report information of *“having received a medical diagnosis of diabetes or high blood sugar” (Yes / No)*, as well as the year of diagnosis in case of an affirmative response. We also included information on age at the survey time (completed years), sex (female / male), socioeconomic status (SES, household index constructed from variables of material capital and human capital, collected by each survey), and smoking habit (never smoker, ex-smoker, and current smoker). During the survey visit subjects were weighed and their height measured; using this information we classified BMI (kg/m^2^) in 3 categories: normal (< 25), overweight [25–29] and obesity (> 29). We excluded subjects who did not have information of self-report on medical diagnosis of DM, with a diagnosis of gestational DM, without information from the year of diagnosis, or who were diagnosed before the age of 20 (criterion to differentiate type 2 diabetes from type 1 diabetes [46]).

### PM_2.5_ exposure information

We estimated PM_2.5_ exposure at a residential block level, using two methods, one based on ground monitor data (*“monitors”*), the other using satellite data (*“satellite”*).

### Monitors method

The monitors method was developed based on PM_2.5_ data obtained from the atmospheric monitoring network of Mexico City, dating from 2004 to 2012 with spatial and temporal coverage extended through PM_2.5_ / PM_10_ ratio estimation methods: We calculated hourly PM_2.5_ / PM_10_ ratios and their median ratios grouped by hour of the day, day of the week, weather season and year (insufficient spatial variability was detected). With these median ratios, we estimated missing hourly PM_2.5_ concentrations in monitoring stations measuring PM_2.5_ and PM_10_ simultaneously, and only PM_10_ by multiplying the corresponding available PM_10_ hourly data by the median ratio. From these data, we calculated annual PM_2.5_ averages for each monitoring station and then we estimated the PM_2.5_ annual average concentration of the residential block for each of our study participants. The estimation procedures were carried out through geo-processes of spatial analysis and interpolation through inverse distance weighted (IDW): first, we generated a buffer of 5 km around the monitoring stations that had PM_2.5_ information. We selected the blocks located within the buffer intersection areas and estimated PM_2.5_ concentration using IDW. To estimate the concentration of the missing blocks we repeated the process with 10 km buffers. For blocks located within the 10 km buffer but outside the intersections (i.e both of the 5 and 10 km buffers), we assigned the average annual PM_2.5_ concentration recorded for the buffer monitoring station in which it was located. The PM_2.5_ concentration in blocks that did not meet any of the previous criteria was estimated using the IDW using the values ​​of all monitoring stations. Our constructed method allowed information from 2 to 3 other monitoring stations to influence the exposure assigned to each subject in cases where the 5 or 10 km circles around additional monitors overlapped the subjects’ residence location.

### Satellite method

This method was developed based on satellite remote sensing measurements of AOD, a quantitative measure of the amount of particles in an atmospheric column in a 1 × 1 km grid. AOD measurements are taken daily at 2:57 p.m. (Greenwich Mean Time (GMT) - 5 h; range 2:10–3:45 p.m., local flyover time), and are derived from the MODIS Aqua satellite (Collection 6 L1B). The model estimates PM_2.5_ concentrations in the MCMA since 2004 and was adjusted and calibrated using a multi-step land use regression modeling (LUR) approach, including AOD values and information measured by the MCMA air quality monitoring system as described in detail by Just and collaborators [50].averages were calculated by applying the criterion of 75% completeness of measurement series in a day or year. Other spatial and temporal predictors of PM_2.5_ considered were roadway density, meteorological data (temperature and relative humidity), planetary boundary layer and daily precipitation. Annual averages were aggregated by residential block for each of our study participants. When satellite data were missing, AOD estimates were predicted using spatial and temporal smoothing. The model had an out-of-sample cross validation R^2^ of 0.724. We used R, version 1.1.442 (R Core Team) for both exposure estimation methods.

### Cross-sectional studies

In this methodological approach, we analysed data from ENSANUT 2006 and ENSANUT 2012 as two independent cross-sectional studies. From the 2006 data, we excluded subjects for lack of self-report information of medical diagnosis of DM (*n* = 225), lack of information on diagnosis year (*n* = 1), DM diagnosed younger than 20 years old (*n* = 2) and women with a gestational diabetes diagnosis (*n* = 3). For 2012 we excluded subjects for lacking information on the year of diagnosis (*n* = 3) and those diagnosed before the age of 20 (*n* = 5). No subjects were excluded due to incomplete exposure information.

For the statistical analysis we used logistic regression models adjusted for sex, age, SES and smoking, weighted for the population of the study area with independent expansion factors of each subsample. For each of the exposure estimation methods (monitors and satellite), we calculated the prevalence odds ratio with 95% confidence intervals (95% CI). The analyses were carried out using the *svy* command for survey analysis in Stata, version 14.

### Case - control study

Cases were defined as subjects ≥20 years old who reported “*having received a medical diagnosis of diabetes or high blood sugar*”. We excluded cases for not having information of the year of DM diagnosis (*n* = 3), and who were diagnosed with DM < 20 years old (*n* = 5). The controls corresponded to adults in the MCMA ENSANUT 2012 without a diagnosis of DM. To improve statistical efficiency, cases and controls were matched 1:4 by age (+ 1 year) and sex without replacement. No subjects were excluded due to incomplete exposure information.

The relationship was analysed using conditional logistic regression models, adjusted by SES and smoking status. We calculated the odds ratio with 95% CI for each of the exposure estimation methods. We performed graphical and numerical tests of goodness of fit for choosing the best explanatory model of both approaches and evaluated the potential modification effect by the adjustment variables. Due to the matching, the analyses were not adjusted by the survey design weights.

For both analytic approaches we considered BMI could be an intermediate variable in the relation between PM_2.5_ and DM, therefore we performed our main analysis excluding it from our models. Nonetheless we evaluated models adjusting for BMI and a possible effect modification in stratified analyses. Analyses were carried out using Stata, version 14.

## Results

The population characteristics of the cross-sectional studies are presented in Table 1. The prevalence of DM was 8.3% (95% CI: 6.9–9.8) for subjects in the ENSANUT 2006, and 11.3% (95% CI: 10.3–12.6) for the ENSANUT 2012. The average age in 2006 and 2012 was 42 years, and subjects with DM were significantly older than those without DM (58 vs. 41 years, *p* < 0.01). SES was also significantly higher in people with DM in both years. Smoking habit in 2006 was similar among people with and without DM, while in 2012 it was significantly higher in people without DM. Obesity was more prevalent in people with DM (2006 prevalence = 37.0% in people with DM vs 30.1% in people without DM, 2012 prevalence = 35.0% in people with DM vs 30.3% in people without DM). The annual average of satellite PM_2.5_ exposure was 25.0 ± 0.2 μg/m^3^ among people with DM and 24.8 ± 0.2 μg/m^3^ among people without DM, a very small difference that was statistically significant (Table 1). Levels of PM_2.5_ exposure in the year previous to the ENSANUT surveys estimated with monitors and satellite were higher in 2006 than in 2012 (Table 2).

**Table 1:** Descriptive statistics of the study participants from ENSANUT surveys 2006 and 2012

	2 0 0 6					2 0 1 2				
	With Diabetes		Without Diabetes		*p value*	With Diabetes		Without Diabetes		*p value*
	n	% (CI 95%)	n	% (CI 95%)		n	% (CI 95%)	n	% (CI 95%)	
Sample size	193	8.3	2082	91.7		284	11.3	2013	88.7	
Weighted sample size	1,045,037	(6.9–9.8)	11,610,723	(90.1–93.1)		1,547,262	(10.3–12.6)	12,184,640	(87.3–89.8)	
Age in years(mean, SE)	57.9	1.5	40.7	0.5	*0.00*	57.7	1.2	40.9	0.5	*0.00*
Sex, men (n, %)	515,155	49.3	5,169,931	44.5	*0.30*	719,601	46.5	5,578,789	45.8	*0.85*
Socioeconomic status (mean, SE)	0.0	0.1	−0.2	0.1	*0.05*	0.0	0.1	−0.1	0.1	*0.05*
Body Mass Index (n, %)										
Normal (< 24.9)	167,535	21.3	2,153,242	28.4		273,960	24.8	2,283,617	28.8	
Overweight (25.0–29.9)	329,019	41.7	3,147,836	41.5		444,872	40.2	3,247,347	40.9	
Obesity (> 30.0)	291,422	37.0	2,287,117	30.1	*0.14*	388,005	35.0	2,408,519	30.3	*0.44*
Smoking status (n, %)										
Never	462,300	44.2	5,208,715	44.9		783,924	50.7	4,384,240	36.0	
Former	122,893	11.8	1,872,923	16.1		202,178	13.0	2,017,408	16.6	
Current	459,844	44.0	4,529,085	39.0	*0.26*	561,160	36.3	5,777,546	47.4	*0.00*
PM_2.5_ previous annual average (mean, SE)										
Monitor	26.8	0.3	27.2	0.4	*0.15*	24.4	0.5	24.4	0.4	0.79
AOD	26.2	0.3	26.1	0.2	*0.31*	25.0	0.2	24.8	0.2	*0.02*

**Table 2:** Summary statistics on exposure to PM_2.5_ (μg/m^3^) during the year previous to ENSANUT survey

Year	Measuring method	Mean **+** SD	Percentiles					
			Min	25th	50th	75th	Max	IQR
*2006*	*Monitor*	26.9 + 3.2	19.3	25.8	26.9	28.2	34.8	2.3
	*Satellite*	26.1 + 2.4	21.4	24.3	26.0	27.9	30.4	3.6
*2012*	*Monitor*	24.1 + 3.5	20.3	21.3	23.2	25.0	31.9	3.7
	*Satellite*	24.8 + 1.5	21.3	23.7	24.8	26.2	27.9	2.5

### Results of the cross-sectional studies

The 2006 study showed no association between PM_2.5_ exposure and DM in crude and adjusted models for either exposure estimation method. However, for the 2012 study, we observed that an increase of satellite estimated 1 μg/m^3^ PM_2.5_ was associated with 12% increased odds of DM and a 3 fold increase in the odds of DM associated with a 10 μg/m^3^ increase of PM_2.5_, in models adjusted for age, sex and smoking (Table 3). We did not observe effect modification by sex, BMI, smoking, or SES in any model.

**Table 3:** Odds Ratios of DM associated with a unit and 10 μg/m^3^ increase of PM_2.5_, measured by monitor and AOD. ENSANUT 2006 and 2012

Year	PM_**2.5**_ measure	Adjusted for age, sex and SES		Additionally adjusted for smoking	
		OR	95% CI	OR	95% CI
2006	**Monitor**				
	per 1 μg/m^3^ increase	0.95	(0.90–1.00)	0.95	(0.90–1.00)
	per 10 μg/m^3^ increase	0.62	(0.36–1.07)	0.61	(0.35–1.04)
	**AOD**				
	per 1 μg/m^3^ increase	0.95	(0.88–1.03)	0.96	(0.88–1.04)
	per 10 μg/m^3^ increase	0.63	(0.27–1.43)	0.66	(0.29–1.50)
2012	**Monitor**				
	per 1 μg/m^3^ increase	1.01	(0.96–1.05)	1.00	(0.95–1.04)
	per 10 μg/m^3^ increase	1.10	(0.72–1.70)	1.02	(0.65–1.60)
	**AOD**				
	per 1 μg/m^3^ increase	1.08	(0.98–1.18)	**1.12**	**(1.01–1.23)**
	per 10 μg/m^3^ increase	2.16	(0.87–5.36)	**3.09**	**(1.17–8.15)**

### Results of the case-control study

The study population consisted of 121 cases and 480 controls, with a mean age of 51 ± 13 years, and 40% were men. Cases had higher SES than controls, a higher prevalence of obesity, and a higher prevalence of never smokers (Table 4). The average PM_2.5_ exposure two years prior to case diagnosis was 24 μg/m^3^ on average, estimated with either monitors or satellite (Table 5).

**Table 4:** Population characteristics for the case-control study, using data from ENSANUT 2012

	Cases		Controls		
	*n* = 121		*n* = 480		
	Mean	SD	Mean	SD	*p value*
Age	51.7	13.4	51.3	13.0	*0.79*
Sex, men (n, %)	49	40.5	196	40.8	*0.95*
Socioeconomic status (mean, SD)	0.2	1.1	−0.1	1.0	*0.00*
BMI (n, %)					
Normal (< 24.9)	11	12.4	72	20.4	
Overweight (25.0–29.9)	33	37.0	154	43.6	
Obesity (> 30.0)	45	50.6	127	36.0	*0.04*
Smoking status (n, %)					
Never	58	47.9	189	39.4	
Former	28	23.2	86	17.9	
Current	37	28.9	205	42.7	*0.02*
PM_2.5_ exposure two years before cases diagnosis (mean, SD)					
Monitor	24.4	4.0	24.4	4.3	*0.89*
AOD	24.2	2.2	24.1	2.3	*0.62*

**Table 5:** Summary statistics on PM_2.5_ (μg/m^3^) 2 years before cases diagnosis

Measuring method	Mean **+** SD	Percentiles					
		Min	25th	50th	75th	Max	IQR
*Monitor*	24.4 + 4.3	15.8	21.5	23.6	26.7	36.9	5.2
*Satellite*	24.1 + 2.3	19.1	22.5	24.0	25.6	29.9	3.1

Exposure to PM_2.5_ measured with monitors was not associated with DM. Using satellite measures we observed a positive association, however it was not statistically significant. In Table 6 we show the OR of the crude and adjusted models by age, sex, SES and smoking. We did not observe differences in the OR in models with and without adjustment by BMI nor did we observe effect modification by BMI, smoking or SES.

**Table 6:** Case - control study using ENSANUT 2012 data. Odds Ratios (95% confidence intervals) of PM_2.5_ exposure two years prior to cases diagnosis and DM

PM_**2.5**_ exposure	Crude		Adjusted for age, SES and smoking	
	OR	95% CI	OR	95% CI
**Monitor**				
Per 1 μg/m^3^	1.00	(0.95–1.05)	0.99	(0.94–1.04)
Per 10 μg/m^3^	0.96	(0.57–1.60)	0.90	(0.53–1.52)
**AOD**				
Per 1 μg/m^3^	1.03	(0.92–1.16)	1.10	(0.97–1.25)
Per 10 μg/m^3^	1.36	(0.42–4.40)	2.55	(0.70–9.21)

## Discussion

To our knowledge, this is the first study to evaluate the relationship between exposure to PM_2.5_ and DM in Mexico, a middle-income country in the Americas and in a representative sample of the MCMA; it is pioneer in this mega-urban area and considers a representative subsample of the adult population, two epidemiological approaches, and two different exposure methods The results of the cross-sectional study in 2012 suggest that there is a positive association between PM_2.5_ exposure and DM in the population of the MCMA, particularly when the exposure is estimated from satellite information; these results are reinforced by the satellite exposure case-control results, where we observed positive albeit not statistically significant associations.

Intriguingly, the cross-sectional results for the ENSANUT 2006 are not in the same direction as those from 2012. This could be explained since the exposure information prior to 2011 has a greater degree of inaccuracy with respect to that year and beyond. This is reported in more detail in a previous study by our research group [51]. Briefly, the air quality monitoring system had fewer monitoring stations of PM_2.5_ before 2011. Between 2004 and 2010 only 3 monitoring stations measured both PM_10_ and PM_2.5,_ this increased to 7 in 2011 for a maximum of 12 in 2016. Both exposure methods, monitors and satellite, were calculated and / or calibrated with such available information. For the monitors approach, we used a PM_2.5_ / PM_10_ ratio estimation method that had correlations between the observed and predicted values of 0.60–0.84 from 2004 to 2010 and above 0.90 from 2011 onward. We consider that including the results of the full data analyses (2006 and 2012) is important, despite the possible limitations, since future studies might consider this research question and should be able to compare their results with this work.

The inconsistency of the results in our study is similar to that observed in cohort studies [10, 12, 13, 17] and in cross-sectional studies [15, 32], where results observed suggest positive associations, but without conclusive results. Some of the methodological coincidences that could be related to what we observed are: the sample size, the method of exposure estimation, the variability of PM_2.5_ exposure concentrations, and the method of DM determination. Our study used data of self-reported DM diagnosis, this could be largely underestimated considering the amount of people in Mexico living with undiagnosed DM. This could potentially have biased our results to the null, therefore future studies should, ideally, include laboratory tests for DM diagnosis.

Studies that have reported concentration-response functions of PM_2.5_ exposure have observed a significant increase in the risk of DM with exposure to the first 10 μg/m^3^, then a “plateau” where the risk increase is much smaller, compared to the first micrograms of exposure [52, 53]. In our study, the average exposure was similar between monitor and satellite measures in the two study designs, and in all cases, it was twice the maximum permissible limit established in the Official Mexican Standard (12 μg/m^3^), as well as the recommended by WHO (10 μg/m^3^) and US EPA (12 μg/m^3^). Likewise, the minimum exposures were around 20 μg/m^3^ and the maximum ones 30 μg/m^3^. This information suggests that our entire study population was exposed to elevated concentrations, without an exposure gradient with sufficient variability to compare and detect the effects at lower concentrations or in a non-linear function [15].

The proxy to personal exposure estimate implies an inherent measurement error that could influence the results observed in our study. For comparative purposes with other studies and among our sources of PM_2.5_ information (monitors and satellite), in this study we used PM_2.5_ concentrations at residential block level as an estimate of personal exposure. Our retrospective exposure approaches consisted of the average exposure of the year prior to the survey in cross-sectional analyses, under the assumption of similar and constant exposure in previous years. In the case-control approach, we used the two-year average prior to the year of cases DM diagnosis and their respective controls. These approaches could have underestimated the actual exposure since they do not consider individual differences, the activity and mobility patterns, or residential changes. The estimation of exposure in the home, under the assumption of being constant over time, and not varying with activities, adds uncertainty to the estimation of the exposure [33] and does not allow analyzing differences related to these mobility patterns, such as those observed between men and women in other studies [5]. However, measurement error was non-differential between the groups with and without DM.

We could not account for particle composition or other PM_2.5_ sources. It has been previously observed that there are variations in PM_2.5_ particle composition, in time and space in the study area [54], and we cannot rule out that these differences could influence the effect on DM, suggesting that the results observed in other regions of the world could be attributable to these variations [15, 31, 40]. According to Vega and collaborators, PM_2.5_ composition depends largely on the type and use of land, most of the sources of PM_2.5_ are automotive, and whose fuels vary in quality and composition depending of the region [54]. Therefore, it is desirable that subsequent studies include information on the composition of PM_2.5_, in order to estimate the association of these components with the pathophysiological mechanisms of DM. Our cross-sectional analyses were carried out using a representative sample of the adult population of the MCMA, however, the width of our confidence intervals suggests that our sample size could have been insufficient.

With respect to adjusting meteorological variables (e.g., temperature and relative humidity), although these variables change on an hourly basis throughout all the days in the one or two year period preceding the date of sampling each subject, we used an average of PM_2.5_ over those periods. PM_2.5_ concentrations vary according to temperature, humidity, and wind conditions and adding a yearly average of those meteorological variables would only partially duplicate what is already measured in the two methods of PM_2.5_ determinations. There isn’t sufficient published information to suggest that meteorological conditions directly affect the development of DM except as they may affect conditions more directly associated with DM. We had limited information on potential confounders thus increasing the possibility of residual confounding. Potential effect modifiers were also missing from our data: environmental (eg. noise, walkability, greenery, and other contaminants), as well as personal (eg. family history of DM, physical activity and consumption of certain foods). The modifying effects of exposure to noise, the greenness of the environment, and other environmental variables are increasingly documented [14, 17, 40], as well as the potential protective effect of food consumption such as fruits [14]. We were unable to analyze if PM_2.5_ had an association with high sugar intake or with reduced physical activity, however a recent publication from our group found increased odds of obesity associated to PM_2.5_ exposure (pooled OR = 1.96 (95% CI: 1.21, 3.18)), with the results for adults suggestive of an association with a pooled OR = 1.50 (95% CI: 0.58, 3.88) and OR_2012_ = 2.73 (95% CI: 0.97, 7.71) [55]. We considered that BMI could be an intermediate variable in the relation between PM_2.5_ and diabetes and did not adjust our final models for BMI, furthermore, our results did not change when adjusting for BMI. Finally, PM_2.5_ data from the two exposure assessments (monitors and AOD) was available between 2004 and 2014 and in this time period only two ENSANUT surveys were carried out (2006 and 2012). Future studies should expand the exposure models to more recent years and use data from more recent ENSANUTs.

We suggest follow-up studies that tackle the methodological limitations described in our study and that provide comparative information of population groups exposed to different concentration levels of PM_2.5_, particularly in populations where chronic diseases such as DM are increasing.

## Conclusions

This study is the first to analyze the association between PM_2.5_ exposure and diabetes mellitus in a representative sample of the Mexico City Metropolitan Area. Our results add to the epidemiological evidence of the association between PM_2.5_ exposure and DM however, the results are still not conclusive. Studies in low and middle-income countries, where PM_2.5_ atmospheric concentrations exceed WHO standards, are required to strengthen the evidence.

## Data Availability

The National Nutrition and Health Surveys (ENSANUT), 2006 and 2012 data are available upon request at https://ensanut.insp.mx/ Data for PM 2.5 exposure can be made available through Dr. Allan Just upon reasonable request.
